# Supplementary material for: Genetic linkage disequilibrium of deleterious mutations in threatened mammals
Source: EMBO Rep. 2024 Nov 1;25(12):5620–34. doi: 10.1038/s44319-024-00307-2 (PMC11624202; doi:10.1038/s44319-024-00307-2)
Supplement: Supplementary file 1 — Appendix [file 44319_2024_307_MOESM1_ESM.pdf]

Appendix for

Genetic linkage disequilibrium of deleterious mutations in threatened mammals

Table of contents:

Appendix Figure S1 .... Page 2

Appendix Figure S2 .... Page 3

Appendix Figure S3 .... Page 4

Appendix Figure S4 .... Page 5

Appendix Figure S5 .... Page 6

Appendix Figure S6 .... Page 7

Appendix Figure S7 .... Page 8

Appendix Figure S8 .... Page 9

Appendix Table S1 .... Page 10

Appendix Table S2 .... Page 11

Appendix Table S3 .... Page 12

Appendix Table S4 .... Page 12

Appendix Table S5 .... Page 12

Appendix Table S6 .... Page 12

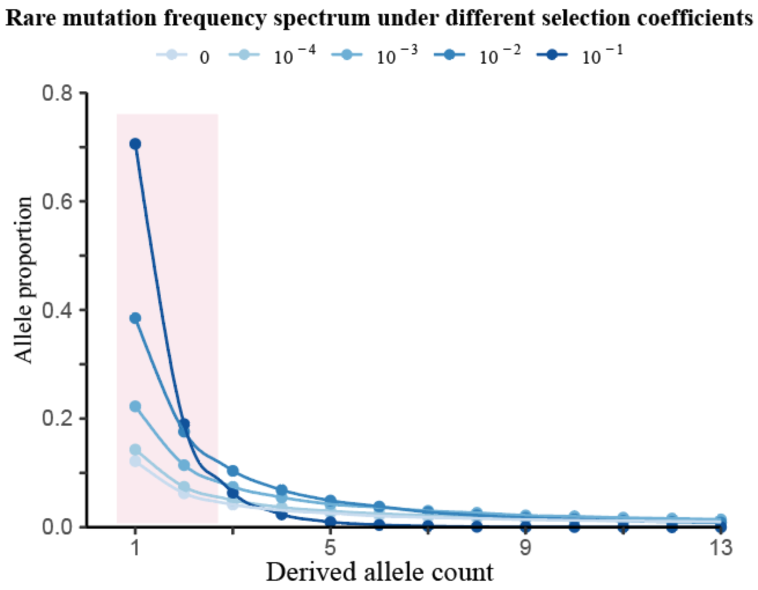

Appendix Figure S1: Doubletons in a sample of 1000 individuals are more deleterious than variants with higher frequency.

We simulated 120 replicates of a constant population size of 14, 474 diploid individuals with r = 1 x 10^{-8} per bp, and genome structure defined in Materials and Methods. As negative selective pressure growths, low frequency variants are of larger proportion. Although simulations predict singletons (first point of each line), on average, should be the most deleterious in our samples, doubletons (second point) are also relatively deleterious. Higher frequency variants tend to have mean selection coefficients that are much more neutral. Because we want to study the effects of negative selection on LD, we restrict many of our analyses to low frequency variants.

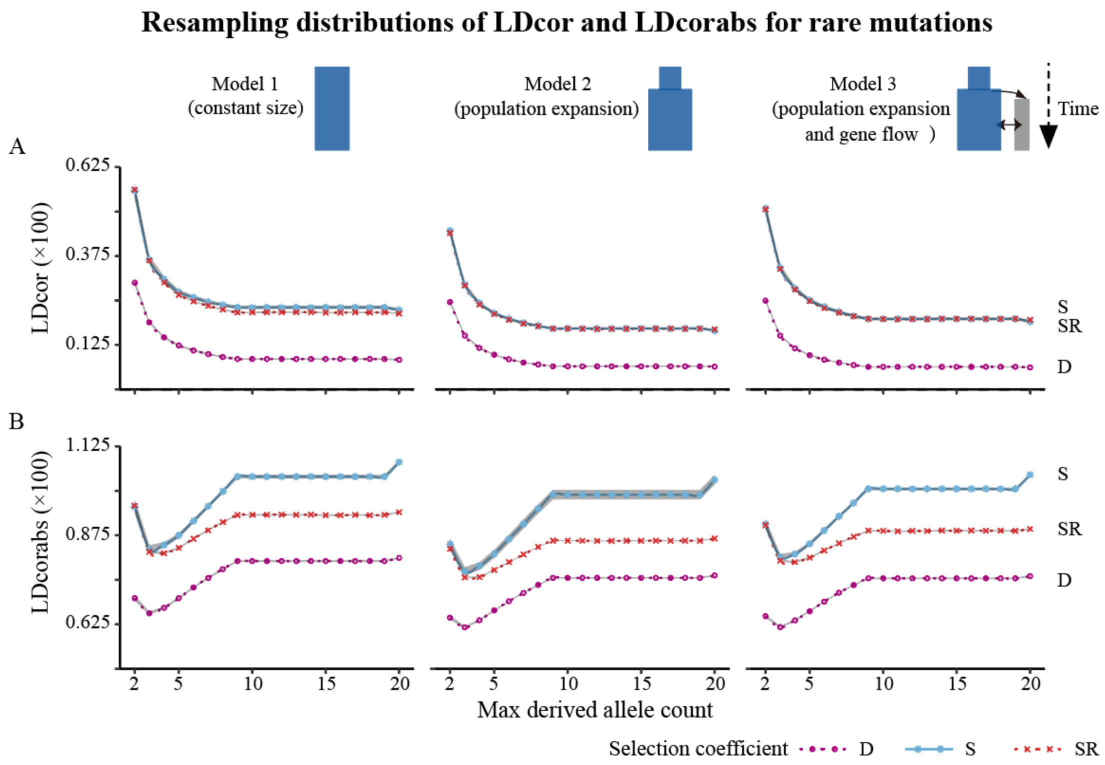

Appendix Figure S2: Mean LDcor and LDcorabs values with different selection coefficient when allele frequency spectrum is controlled.

(**A**) We resampled variants from the 0 simulations with the same allele frequency spectrum of -0.01 simulations and compared LDcor. The blue solid line with represents LD of 0 simulations (named as S), the purple dotted line with dot represents -0.01 simulations (named as D), and the red dotted line with "×" represents 0 simulations but with the same allele frequency spectrum of -0.01 simulations (named as SR). The LDcor of SR is always between that of S and D.

(**B**) The LDcorabs values in S, D, and SR under varying max derived allele count.

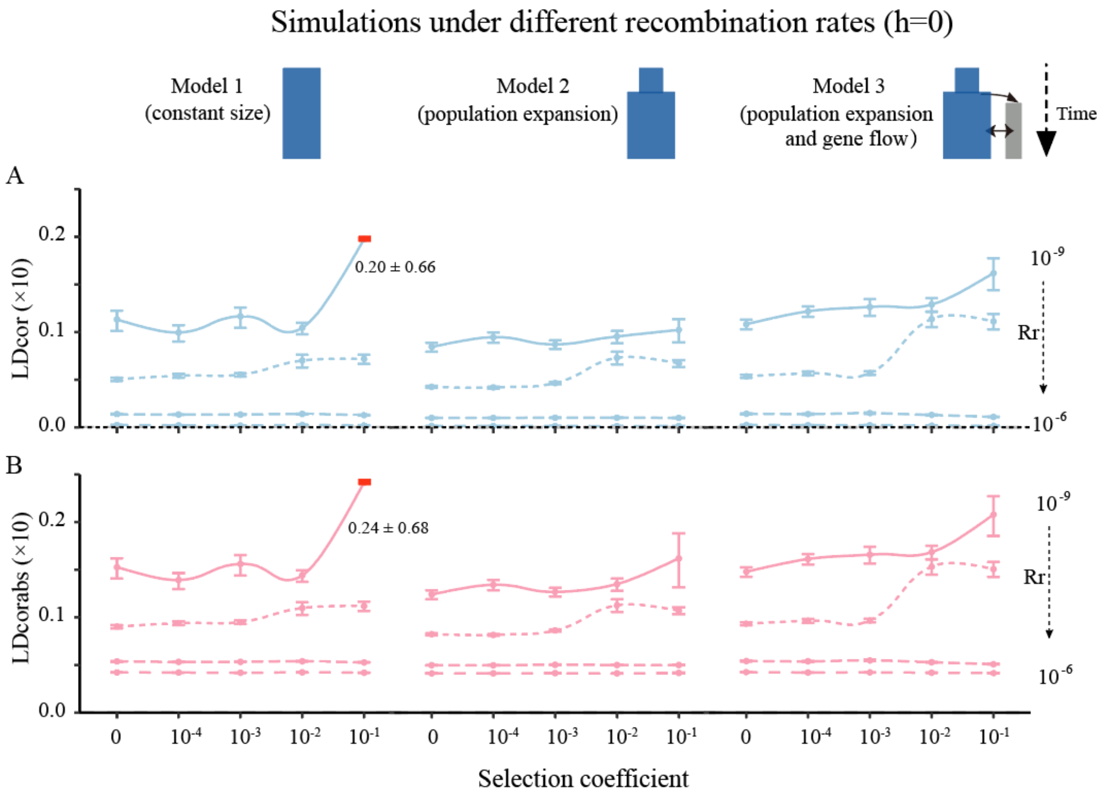

Appendix Figure S3: LDcor and LDcorabs for neutral SNPs with different selection coefficients, recombination rates, and models (h=0.0).

(**A**) LDcor values in three models (model1: constant population size; model 3: with population expansion and gene flow; model 2: with population expansion). We ran 120 simulations for each recombination rate (Rr, values in 10^{-9}, 10^{-8}, 10^{-7}, or 10^{-6}) and each selection coefficient. The point and the error bar represent mean value and standard error mean (s.e.m.) of LDcor.

(**B**) LDcorabs values in three models, we ran 120 simulations for each recombination rate and each selection coefficient. The point and the error bar represent mean value and s.e.m. of LDcorabs.

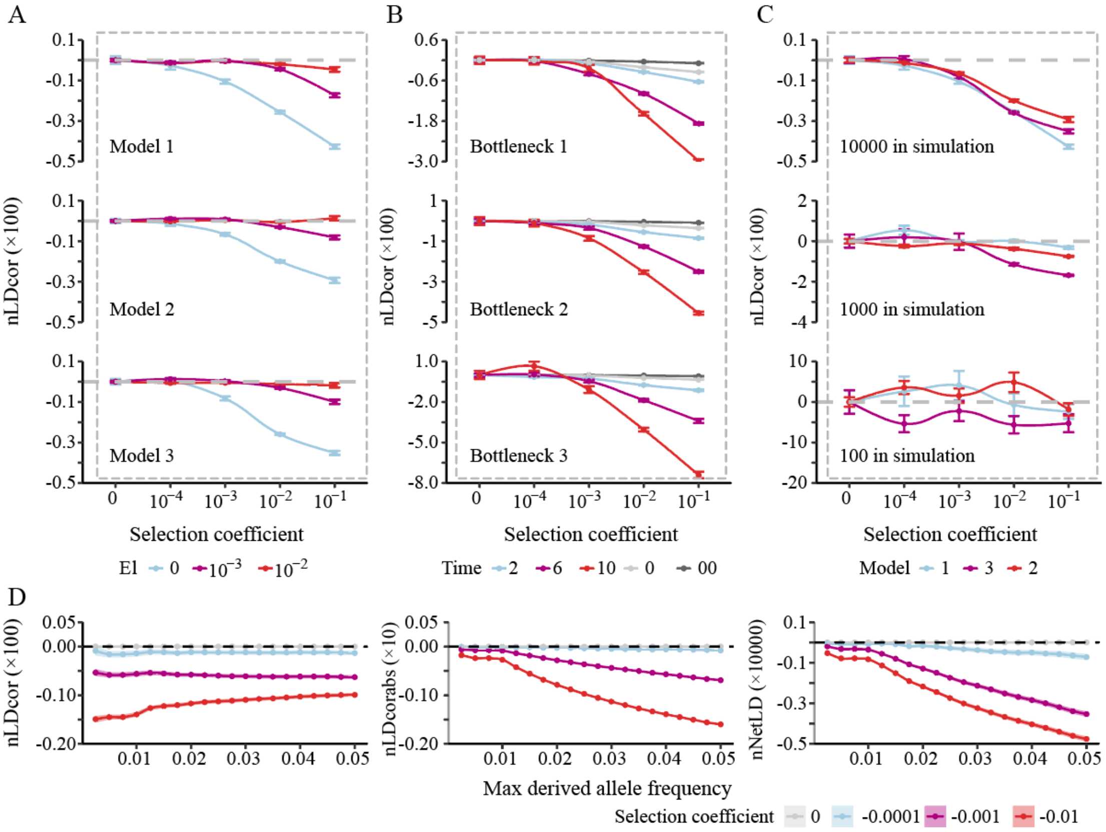

Appendix Figure S4: Simulation of nLDcor with different levels of synergistic epistasis, bottleneck, demography, and max derived allele frequency.

(**A**) We simulated different synergistic epistasis level (0,10^{-3}, 10^{-2}) as well as different negative selective coefficient (0, 10^{-2}, 10^{-3}, and 10^{-4}) of each model (10000 individuals) with a recombination rate of 1 x 10^{-8} crossovers per base pair per generation.

(**B**) In order to compared the nLDcor of varying magnitudes of bottleneck (based on model 1), we simulated different scaled population size (from 10000 to 1000: bottleneck 1, 700: bottleneck2, and 400: bottleneck 3) as well as different duration time (0, 2, 6, and 10 generations). And the dark gray line is the results of no-resampling (10000) at time 0 (e.g., time 00).

(**C**) We test the effect of different models and demography on nLDcor for different selection coefficient (sample1(10000,1000): a simulation with 10000 individuals and a subset of 1000 individuals in calculating; sample2(1000,1000): a simulation with 1000 individuals and a subset of 1000 individuals in calculating; sample3(100,100): a simulation with 100 individuals and a subset of 100 individuals in calculating).

(**D**) For all three models, we examined the nLDcor of variants with selection coefficients of 0 and beyond a certain distance away from each other (10bp, 100 bp, and 1000 bp). The nLDcor values were normalized by reducing the nLDcor of variants without distance limitation. (nNetLD represents normalized NetLD)

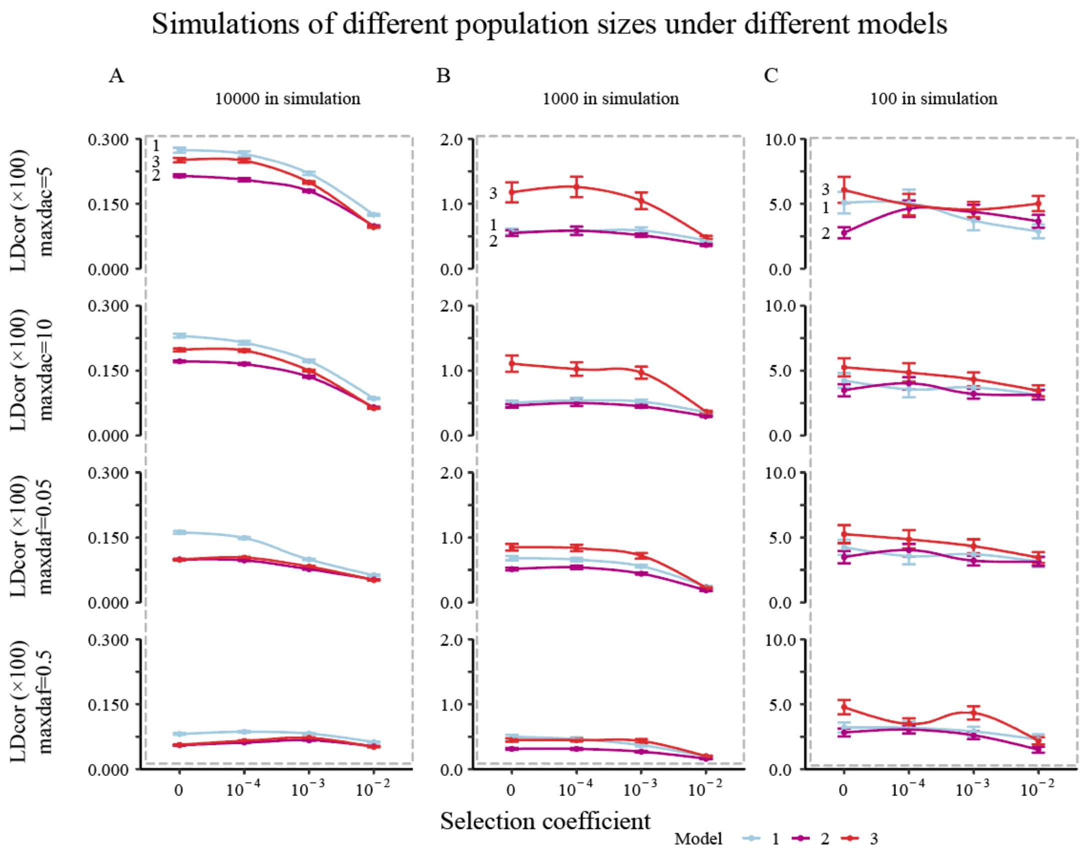

Appendix Figure S5: The effects of different models and demography under different max derived allele counts on LDcor.

Under different derived allele count (dac=5, 10, and 20) and different derived allele frequency (daf=0.05 and 0.5), we test the effect of different models and demography on LDcor for different selection coefficient (sample1(10000,1000): a simulation with 10000 individuals and a subset of 1000 individuals in calculating; sample2(1000,1000): a simulation with 1000 individuals and a subset of 1000 individuals in calculating; sample3(100,100): a simulation with 100 individuals and a subset of 100 individuals in calculating). The points represent mean and the error bars represent s.e.m. of LDcor.

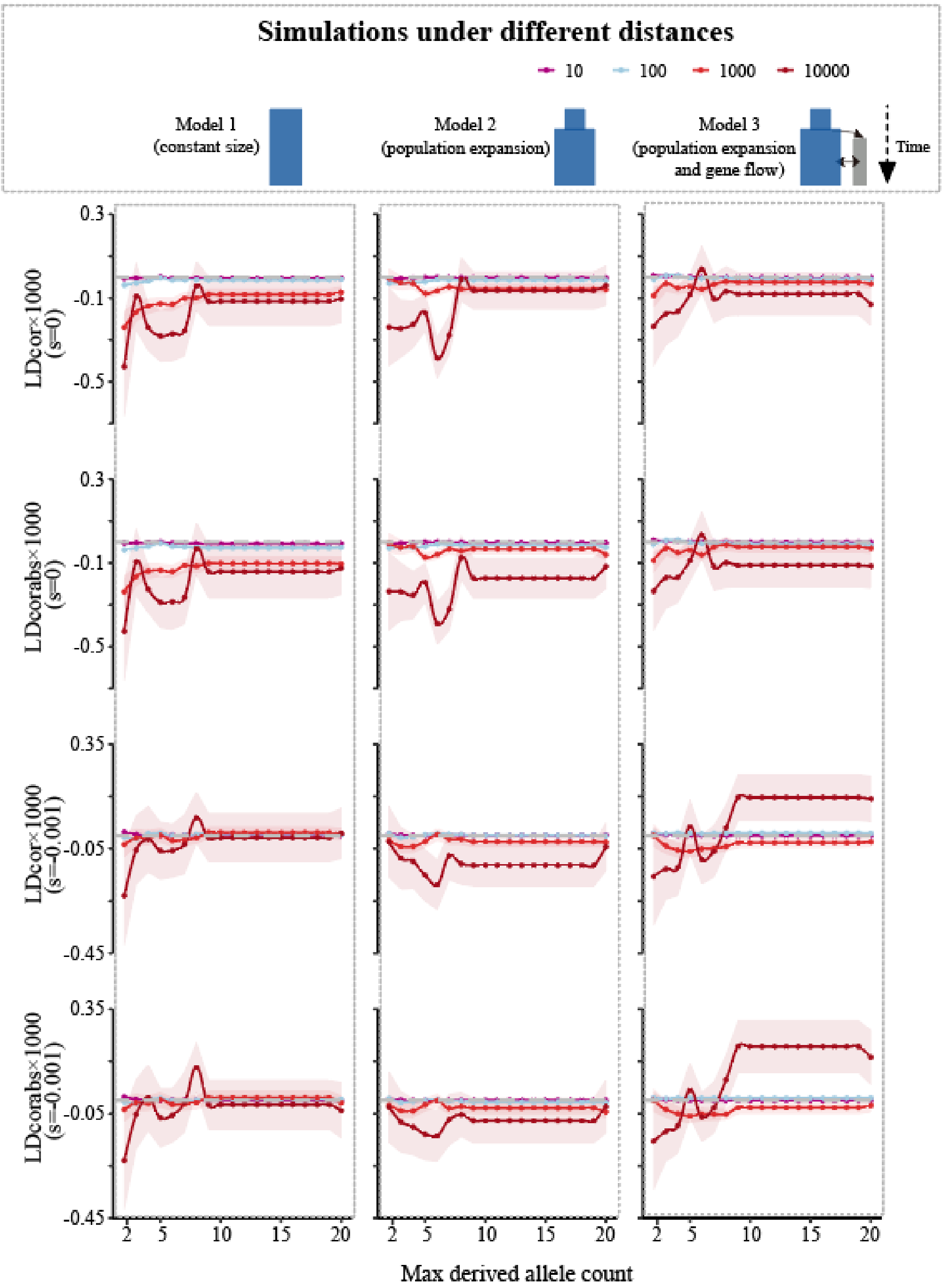

Appendix Figure S6: LDcor and LDcorabs value when only variants beyond certain distance away are considered for different models.

For all three models, we examined LDcor and LDcorabs of variants with selection coefficient 0 (or -0.001) and beyond a certain distance away from each other (10bp, 100 bp, and 1000 bp). The LDcor (LDcorabs) values were normalized by reducing the LDcor (LDcorabs) of variants without distance limitation.

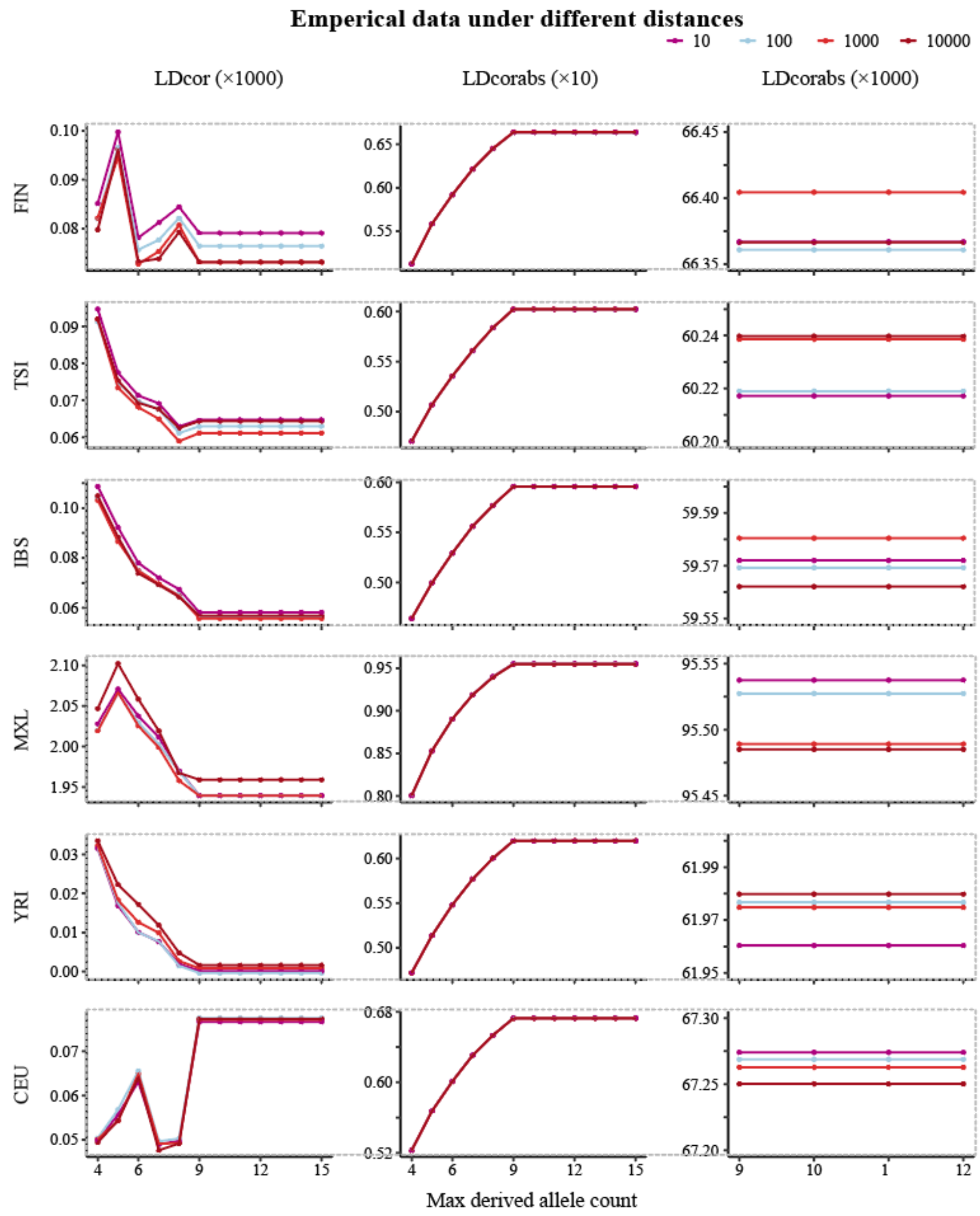

Appendix Figure S7: LDcor and LDcorabs value when only variants beyond certain distance away are considered for multiple populations from 1000 Genomes Project.

To better identify the main source of negative LD, we only take neutral variants beyond a certain distance away from each other into account (10bp, 100 bp, 1000 bp, and 10000 bp). As the null set (variants without distance limitation) overlapped with the 10-bp one (the lightest line), we did not show it.

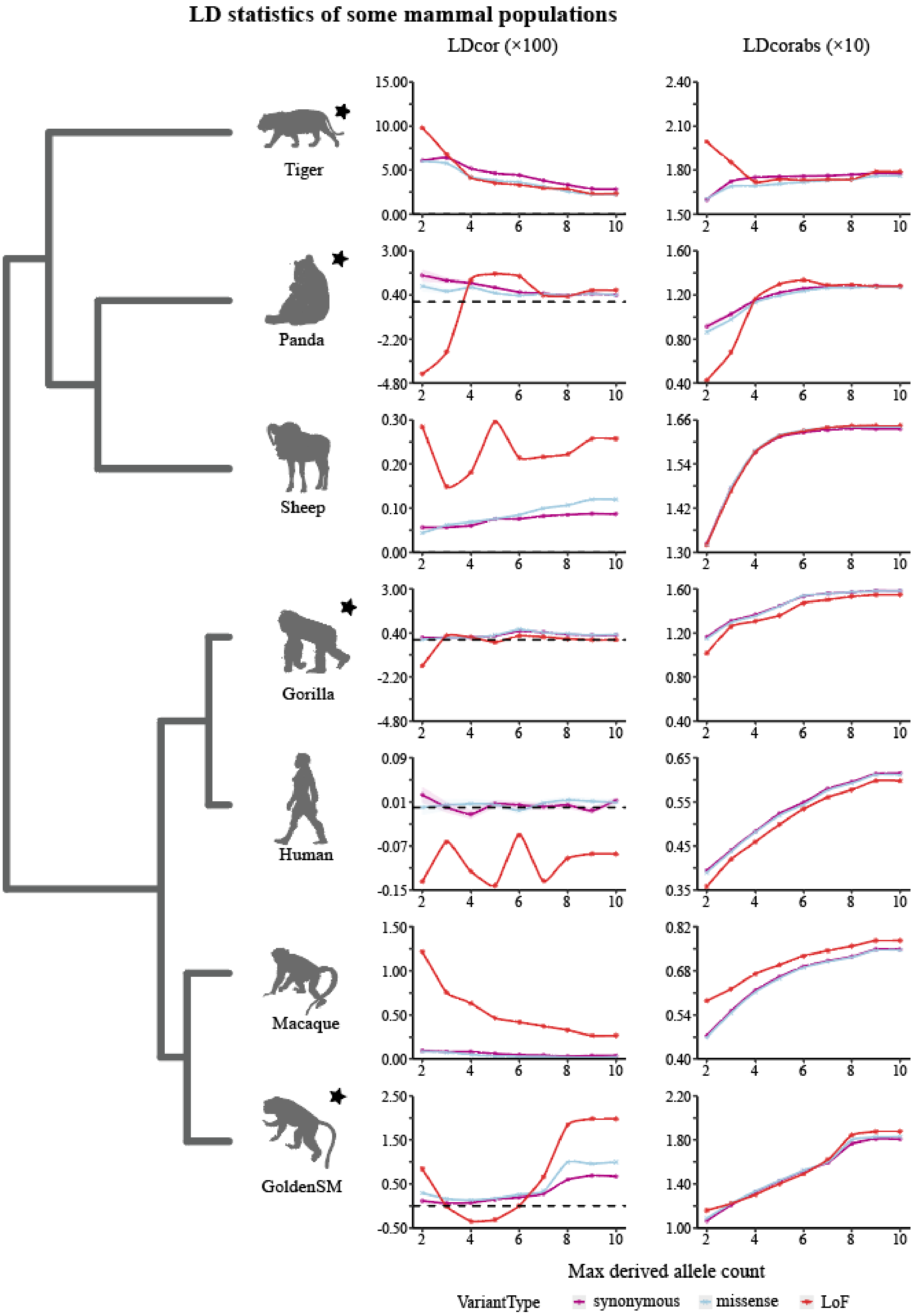

Appendix Figure S8: LDcor and LDcorabs values of empirical data when allele frequency spectrum is controlled.

For CEU (human), macaque, goldenSM (golden snub-nosed monkey), tiger, panda, and sheep, a set of synonymous variants and missense variants with the same allele frequency spectrum of the LoF variants set (the red line) were used for calculation. Each resampling was replicated 1000 times. We then get the distributions of LD values in synonymous and missense variants sets (blue lines indicate median value while shadows indicate the intervals from first to third quartile) with the same allele frequency spectrum of LoF variants set.

Appendix Table S1. LD statistics LDcor and LDcorabs (median) vary with loci distance (MDAC=5).

Model	statistic	Type	10bp	100bp	1000bp	10000bp
constant population size	LDcor	0	0.0026969	0.0026585	0.0025518	0.0023564
		-0.0001	0.0025913	0.0026274	0.0025212	0.0022742
		-0.001	0.0021481	0.0021588	0.0021523	0.0020728
		-0.01	0.0012467	0.0012311	0.0012277	0.0012953
		-0.1	0.0006701	0.0006762	0.0005856	0.0004365
	LDcorabs	0	0.0087551	0.0087010	0.0086105	0.0084350
		-0.0001	0.0086162	0.0086395	0.0085472	0.0082544
		-0.001	0.0080918	0.0081013	0.0081387	0.0080852
		-0.01	0.0069952	0.0069922	0.0069788	0.0070227
		-0.1	0.0054936	0.0055259	0.0054674	0.0053193
with population expansion	LDcor	0	0.0020767	0.0020803	0.0020726	0.0019466
		-0.0001	0.0020373	0.0020106	0.0019549	0.0017088
		-0.001	0.0017950	0.0017773	0.0017229	0.0016465
		-0.01	0.0009619	0.0009580	0.0010049	0.0008309
		-0.1	0.0007317	0.0007317	0.0007135	0.0004434
	LDcorabs	0	0.0080856	0.0080926	0.0080389	0.0078091
		-0.0001	0.0079817	0.0079664	0.0079358	0.0077556
		-0.001	0.0077236	0.0077253	0.0076428	0.0076529
		-0.01	0.0066269	0.0066285	0.0066424	0.0065778
		-0.1	0.0054970	0.0054356	0.0054110	0.0051507
with population expansion and gene flow	LDcor	0	0.0024168	0.0024440	0.0023301	0.0024313
		-0.0001	0.0024075	0.0024154	0.0023827	0.0023168
		-0.001	0.0019929	0.0019855	0.0019202	0.0019222
		-0.01	0.0009621	0.0009389	0.0009404	0.0009821
		-0.1	0.0008920	0.0009112	0.0008425	0.0006206
	LDcorabs	0	0.0084508	0.0084644	0.0083665	0.0085056
		-0.0001	0.0084395	0.0084546	0.0083869	0.0082309
		-0.001	0.0079654	0.0079705	0.0078586	0.0079447
		-0.01	0.0065984	0.0065743	0.0066082	0.0066298
		-0.1	0.0055928	0.0056037	0.0055630	0.0053246

Appendix Table S2. Wilcoxon p value among different loci distance (MDAC=5).

Model	statistic	Type	0_10bp	10bp_100bp	100bp_1000bp	1000bp_10000bp
constant population size	LDcor	0	0.9583741	0.4512872	0.0911634	0.0572317
		-0.0001	0.9583741	0.5119926	0.3001390	0.1166528
		-0.001	0.9583741	0.5154176	0.4099632	0.2667120
		-0.01	0.9583741	0.3941792	0.5000000	0.4572449
		-0.1	0.8961158	0.4854384	0.2500512	0.1564044
	LDcorabs	0	0.9583741	0.4301082	0.0830009	0.0455028
		-0.0001	0.9583741	0.5461608	0.3283255	0.0901117
		-0.001	0.9583741	0.5299579	0.4174928	0.2986470
		-0.01	0.9583741	0.4174928	0.5444584	0.4041296
		-0.1	0.9583741	0.4940029	0.2319889	0.1569198
with population expansion	LDcor	0	0.9583742	0.3503743	0.1157551	0.0970036
		-0.0001	0.9583742	0.4278650	0.2213828	0.0008676
		-0.001	0.9583742	0.5264745	0.1676491	0.1058809
		-0.01	0.9583742	0.5515407	0.6648297	0.0518047
		-0.1	0.8833357	0.5086860	0.4007180	0.1742846
	LDcorabs	0	0.9583742	0.3617345	0.1231339	0.0475614
		-0.0001	0.9583742	0.4049318	0.2074120	0.0005106
		-0.001	0.9583742	0.4674616	0.2557563	0.1111511
		-0.01	0.9583742	0.4743923	0.6544543	0.0900925
		-0.1	0.9583742	0.5104228	0.3666413	0.1472237
with population expansion and gene flow	LDcor	0	0.9583730	0.4707236	0.2209729	0.3629744
		-0.0001	0.9583730	0.4677651	0.2160371	0.1114382
		-0.001	0.9583730	0.5410992	0.0459312	0.4434400
		-0.01	0.9583730	0.3313975	0.4441744	0.7557420
		-0.1	0.9217850	0.5196510	0.5137214	0.1750465
	LDcorabs	0	0.9583730	0.5189099	0.2507229	0.3685699
		-0.0001	0.9583730	0.5226140	0.2215253	0.0666051
		-0.001	0.9583730	0.5440497	0.0614795	0.6151909
		-0.01	0.9583730	0.3491154	0.4751644	0.6467470
		-0.1	0.9583730	0.4951781	0.5144627	0.2344460

Appendix Table S3. The comparison of several LD statistics.

Index	*D*	*D*' / r^2	NetLD	LDcor/LDcorabs	nLDcor
**object**	pairwise loci	pairwise loci	loci set	loci set	loci set
**normalized**	no	yes	no	yes	yes
**directional**	yes	no	yes	yes/no	yes
**comparable between variant sets**	no	yes	no	yes	yes
**comparable between species**	no	no	no	no	yes

Appendix Table S4: The information of genome data from six mammals.

species	name	source	samples
Homo sapiens	Human (CEU)	http://www.1000genomes.org	99
Ailuropoda melanoleuca	giant panda	PRJNA168141	49
Ovis aries	Sheep(AU,MEH）	https://doi.org/10.25919/5d39e494936c6	68
Rhinopithecus roxellana	goldenSM	PRJNA271514	38
Panthera tigris	tiger	PRJNA437782	32
Macaca mulatta lasiotis	macaque	PRJNA345528	32
Gorilla gorilla	gorilla	PRJNA189439	31

Appendix Table S5: The information of ancestral reconstruction for six mammals.

species	reference genome	close species	reference genome	divergence time
Homo sapiens	*hs37d5*	*Gorilla gorilla*	gorGor3	8.60 MYA
Ailuropoda melanoleuca	*ailMel1*	*Canis lupus familiaris*	CanFam3.1	45.1 MYA
Ovis aries	*Oar_v3.1*	*Bos taurus*	ARS_UCD1	21.6 MYA
Rhinopithecus roxellana	*Rrox_v1*	*Macaca mulatta*	Mmul_8.0.1	17.75 MYA
Panthera tigris	*panTig1.0*	*Canis lupus familiaris*	CanFam3.1	55.4 MYA
Macaca mulatta lasiotis	*Mmul_8.0.1*	*Homo sapiens*	hg19	28.82 MYA
Gorilla gorilla	*gorGor3*	*Homo sapiens*	hg19	8.60 MYA

Appendix Table S6. Variant numbers of different functional consequences.

Species	Type	Function	Number
**Tiger**	synonymous	synonymous_variant	16369
	missense	missense_variant	11860
	LoF	stop gained	108

	LoF	stop lost	15
	LoF	splice acceptor variant	38
	LoF	splice donor variant	58
**Macaque**	synonymous	synonymous_variant	7686
	missense	missense_variant	7423
	LoF	stop gained	115
	LoF	stop lost	7
	LoF	splice acceptor variant	24
	LoF	splice donor variant	29
**GoldenSM**	synonymous	synonymous_variant	16893
	missense	missense_variant	19232
	LoF	stop gained	320
	LoF	stop lost	21
	LoF	splice acceptor variant	68
	LoF	splice donor variant	92
**Giant panda**	synonymous	synonymous_variant	3227
	missense	missense_variant	3548
	LoF	stop gained	46
	LoF	stop lost	1
	LoF	splice acceptor variant	6
	LoF	splice donor variant	12
**CEU**	synonymous	synonymous_variant	21637
	missense	missense_variant	27640
	LoF	stop gained	421
	LoF	stop lost	27
	LoF	splice acceptor variant	92
	LoF	splice donor variant	144
**Sheep**	synonymous	synonymous_variant	50742
	missense	missense_variant	44906
	LoF	stop gained	1074
	LoF	stop lost	245
	LoF	splice acceptor variant	313
	LoF	splice donor variant	451
**Gorilla**	synonymous	synonymous_variant	13810
	missense	missense_variant	14295
	LoF	stop gained	196
	LoF	stop lost	8
	LoF	splice acceptor variant	59
	LoF	splice donor variant	73
